# The Role of Soluble Urokinase Plasminogen Activator Receptor (suPAR) as an Early Indicator of Mortality in Pediatric Septic Shock

**DOI:** 10.1002/jcla.25040

**Published:** 2024-05-06

**Authors:** Caner Turan, Ali Yurtseven, Pinar Yazici Ozkaya, Elif Azarsiz, Eylem Ulas Saz

**Affiliations:** ^1^ Division of Pediatric Emergency, Department of Pediatrics Ege University School of Medicine Izmir Turkey; ^2^ Division of Pediatric Intensive Care, Department of Pediatrics Ege University School of Medicine Izmir Turkey; ^3^ Department of Biochemistry Ege University School of Medicine Izmir Turkey

**Keywords:** biomarker, children, sepsis, septic shock, suPAR

## Abstract

**Background:**

Despite advancements in antibiotic therapy and resuscitation protocols, sepsis and septic shock remain major contributors to morbidity and mortality in children. We aimed to investigate the utility of soluble urokinase plasminogen activator receptor (suPAR) for the early detection of septic shock and to evaluate its accuracy in predicting mortality.

**Methods:**

A prospective study was conducted in a tertiary pediatric emergency department (ED), enrolling patients diagnosed with the sepsis, severe sepsis, or septic shock. In addition to assessing infection biomarkers such as C‐reactive protein and procalcitonin, suPAR levels were quantified upon admission using enzyme‐linked immunosorbent assay. The primary outcome measure was 30‐day mortality.

**Results:**

Overall 72 patients and 80 healthy children included. Plasma suPAR levels demonstrated a statistically significant elevation in the sepsis, severe sepsis, and septic shock groups compared with the control group (*p* < 0.001 for all). The septic shock group exhibited the highest suPAR levels upon admission, surpassing both the sepsis and severe sepsis groups (*p* = 0.009 and 0.042). ROC analysis underscored the promising potential of suPAR with an AUC of 0.832 for septic shock. Analysis of mortality prediction revealed significantly higher suPAR levels in nonsurvivors than survivors (9.7 ng/mL vs. 4.2 ng/mL; *p* < 0.001). Employing plasma suPAR levels to discriminate between mortality and survival, a threshold of ≥7.0 ng/mL demonstrated a sensitivity of 90.9% and specificity of 71.0%.

**Conclusion:**

Plasma suPAR levels have the potential as a biomarker for predicting mortality in children with septic shock. In pediatric septic shock, the presence of plasma suPAR ≥7 ng/mL along with an underlying disease significantly increases the risk of mortality.

## Graphical abstract

Despite advancements in antibiotic therapy and resuscitation protocols, sepsis and septic shock remain major contributors to morbidity and mortality in children. Employing plasma suPAR levels to discriminate between mortality and survival, a threshold of ≥7.0 ng/mL demonstrated a sensitivity of 90.9% and specificity of 71.0%. In pediatric septic shock, the presence of plasma suPAR levels might be an ideal biomarker for predicting the associated outcomes and mortality.

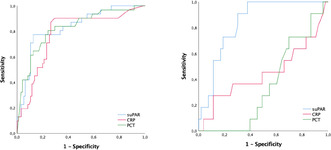

## 1 Introduction

Septic shock is a life‐threatening condition characterized by a dysregulated systemic response to infection, leading to organ dysfunction and mortality [1]. Early recognition and prompt intervention are crucial for improving outcomes in pediatric patients presenting to the emergency department (ED) with sepsis. The identification of reliable biomarkers that can serve as early indicators of septic shock and mortality is of paramount importance in pediatric ED. The accurate prognostication of septic shock and mortality risk in children is crucial for timely intervention and improved clinical outcomes in emergency settings.

Biomarkers, which are biological substances that represent normal or disease‐related processes, can serve as valuable indicators for clinicians. An optimal biomarker designed to identify patients requiring intensive monitoring and treatment should exhibit precision and easy bedside accessibility. Among the biomarkers investigated extensively and utilized in severe sepsis patients, C‐reactive protein (CRP) and procalcitonin (PCT) stand out as the most prominent [2, 3, 4]. The soluble urokinase plasminogen activator receptor (suPAR) has gained attention in recent years [5]. The suPAR is a soluble form of urokinase‐type plasminogen activator receptor that is released into the bloodstream during immune system activation and inflammation. It has been proposed as a potential tool for the risk stratification and early identification of septic shock in adult populations [5].

The main advantage of suPAR as a biomarker is its ability to detect septic shock at an early stage. This early elevation suggests that suPAR could serve as an important tool for identifying high‐risk patients at an earlier stage, enabling timely intervention, and potentially improving outcomes. Studies investigating the role of plasma suPAR levels in the diagnosis of septic shock have predominantly been found in adult literature [6]. This study showed that suPAR levels were elevated even before the onset of clinical symptoms and rise in conventional markers, CRP and PCT [2, 3, 4]. However, in adults, the diagnostic cutoff value has been reported only in cases of Crimean–Congo hemorrhagic fever, which was identified as 3.06 ng/mL [7]. Additionally, serum suPAR levels have been reported to increase in adults with acute kidney injury (AKI), and the increase in suPAR levels may be associated with the severity of AKI [8].

This study aimed to determine the blood levels of suPAR in septic shock and its association with mortality.

## 2 Materials and Methods

### 2.1 Study Design and Setting

This study was conducted in a pediatric ED between March 2018 and June 2020. The ED is an academic tertiary care center with approximately 90,000 visits annually. This study was approved by the Ege University Institutional Review Board (17‐8.1/46), and written informed consent was obtained from all the patients and their parents. To maintain confidentiality, the forms did not include any data that would have enabled the identification of patients. This study was supported by the Scientific Research Projects of the Ege University (ID No.: TTU‐2019‐20230). This trial was registered at Clinicaltrials.gov (No. NCT04459572). The procedures were by the ethical standards of the responsible committee on human experimentation (institutional or regional) and the Helsinki Declaration of 1975.

### 2.2 Sample Size, Definitions, and Patient Selection

Studies examining the levels of suPAR in pediatric populations, particularly focusing on critically ill children, were systematically reviewed in the literature. Subsequently, utilizing the findings from these studies, sample size calculations were conducted [9, 10, 11]. With a confidence level of 95%, a margin of error (confidence interval) of 5%, and assuming a population size of 70, a sample size of 60 was determined to be necessary. Systemic inflammatory response syndrome (SIRS), sepsis, and organ dysfunction were defined by the International Consensus Conference on Pediatric Sepsis and pediatric sequential organ failure assessment (pSOFA) score (Table S1) [12, 13]. SIRS was defined as two or more of the following criteria: fever (>38.5°C) or hypothermia (<36°C), tachycardia (heart rate >2SD) or bradycardia for <1 year (heart rate <10th percentile), respiratory rate >2SD or need for mechanical ventilation, leukocytosis or leukopenia for age, or immature neutrophils >10%. Sepsis is defined as the presence of suspected or proven SIRS infection. If cardiovascular dysfunction, acute respiratory distress syndrome (ARDS), or dysfunction in more than two other organ systems is diagnosed with sepsis, it is described as severe sepsis. Septic shock, defined as cardiovascular dysfunction, does not regress despite a loading of >40 mL/kg of isotonic fluid within an hour [13].

All patients who met the criteria for sepsis, severe sepsis, or septic shock were included in the study. Patients were divided into three groups according to updated guidelines: sepsis, severe sepsis, and septic shock [13].

Children in the same age and the gender group who presented to the general pediatric outpatient clinic were included in the study as the control group. The children included in the control group were those who presented to the outpatient clinic for routine health checkups, without any complaints or illnesses. Children who had underlying diseases or symptoms of infection such as fever, cough, runny nose, sore throat, vomiting, and diarrhea, or who had an infection in the last 10 days or had currently an active infection, or with rheumatic diseases, or immunodeficiency or immunosuppressive drugs use were excluded from the control group.

### 2.3 The Study Protocol, Collection of Blood Samples, and Measurement of suPAR

The patients were analyzed in terms of demographic data (gender, age), main complaints at the time of admission to the ED, clinical characteristics, and diagnoses.

Blood samples were collected by standard venipuncture techniques. Venous blood samples (2 mL each) containing EDTA were promptly obtained from all patients diagnosed with sepsis, severe sepsis, or septic shock in the ED, specifically for measuring plasma suPAR levels. The blood samples were centrifuged for 15 min at 1000 *g*, 2–8°C, within 30 min of collection, and the plasma samples were separated and stored at −80°C until testing. Plasma suPAR levels were measured using a commercially available enzyme‐linked immunosorbent assay (ELISA) kit (Human suPAR ELISA Kit; MyBioSource MBS7606253, USA) according to the manufacturer's instructions. The intra‐assay coefficient of variation (CV) ranged from 4.2% to 5.2%, and inter‐assay CV was 5.1–5.4%. The test was based on sandwich ELISA technology, and the assay range was 0.156–10 ng/mL, with an analytical sensitivity of 0.094 ng/mL to suPAR. According to the kit protocol, 100 μL of plasma samples (1:2 diluted) and standard solutions were added to micro‐ELISA plate wells coated with anti‐suPAR antibodies. After the biotin‐conjugated anti‐suPAR antibody was added, the cells were washed. Horseradish peroxidase (HRP)‐streptavidin conjugate was added to bind suPAR in the sample, and the unbound conjugates were removed by washing. The chromogen tetramethylbenzidine (TMB) substrate was used to visualize the enzymatic reaction. TMB was catalyzed by HRP to produce a blue product that changed to yellow after the addition of an acidic stop solution to terminate the reaction. The density of yellow was proportional to the amount of suPAR in the sample captured on the plate. The optical density was measured photometrically at 450 nm using a microplate reader (Multiskan EX spectrophotometer, Thermo Fisher Scientific, USA). Plasma suPAR concentrations were calculated using a standard curve prepared using standard solutions of eight different concentrations. Samples above the concentration limit of the test were re‐measured after fivefold dilution.

### 2.4 Outcomes

The primary outcome was the determination of suPAR levels for the diagnosis, prognosis, and management of septic shock. The secondary outcome was 7th and 30th day mortality.

### 2.5 Statistical Analysis

The Statistical Package for the Social Sciences (SPSS) software (version 25.0; Armonk, NY: IBM Corp) was used for data analysis. The Shapiro–Wilk test was used to assess the normal distribution of numeric variables before analysis. The continuous variables were given as mean ± standard deviation and median (interquartile range [IQR], min–max), and the categorical variables were given as number and percentage. Mann–Whitney *U* and chi‐square tests were used for comparison. Multivariate and univariate logistic regression analyses were used. Receiver operating characteristic (ROC) analysis was utilized for significant variables to calculate cutoff points, with sensitivity and specificity, positive predictive value (PPV), and negative predictive value (NPV) of the markers. The DeLong and Hanley tests were used to compare CRP, PCT, and suPAR ROC curves among the sepsis, severe sepsis, and septic shock groups, as well as in terms of mortality [14, 15]. On the basis of the ROC analysis and Youden index, which defines the maximum potential effectiveness of a biomarker, the best cutoff points to predict or to exclude septic shock and mortality were selected. Also, Bootstrapping (resampling times = 1000) and Hosmer–Lemeshow statistics were performed for the internal validation and calibration of the model, respectively. Survival analysis was performed using the log‐rank test. Two‐sided tests at *α* = 0.05 level of significance were used for hypothesis controls.

## 3 Results

### 3.1 Demographics

**FIGURE 1:**
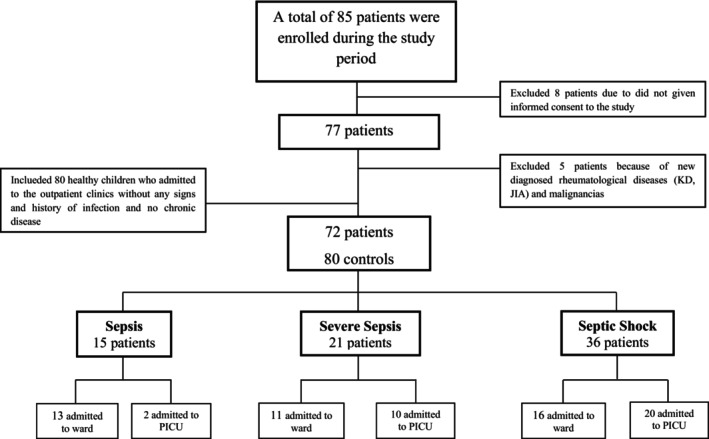
Distribution of patients enrolled in the study period.

Overall 158,560 patients were admitted to the ED during the study period. A total of 85 patients were enrolled in the study. Thirteen patients and/or their parents were excluded from the study (eight patients did not given informed consent, and five patients had new diagnosed rheumatological diseases and malignancies). The final analyses were performed on 72 patients and 80 healthy children (control group) (Figure 1). The median age was 4.0 years (min 1 month–max 17.0 years, IQR 1.0–8.0 years), and the male:female ratio was 1:1 in the patient group (Table 1).

**TABLE 1:** Demographics, symptoms, and clinical characteristics of patients (survivors and nonsurvivors) on admission to the ED.^a^

Characteristics	Total	Survivors	Nonsurvivors	*p*
Patients, *n* (%)	**72**	**61** (84.7)	**11** (15.3)	—
Controls (*n*, %)	**80**	—	—	
Age, years, median (IQR)				
Patients	**4.0** (1.0–8.0)	**4.0** (1.3–7.0)	**6.5** (0.7–12.0)	0.796
Control	**3.0** (11 m–8.0)	**3.0** (11 m–8.0)	—	
Gender (F/M)				
Patients	36/36	31/30	5/6	0.743
Control	39/41	39/41	—	
Admission time, *n* (%)				
On working hours	32 (44.4)	27 (44.3)	5 (45.5)	0.942
Out of working hours	40 (55.6)	34 (55.7)	6 (54.5)	
Symptoms, *n* (%)				
Fever	59 (81.9)	52 (85.2)	7 (63.6)	0.086
Respiratory distress	22 (30.6)	14 (22.9)	8 (72.7)	**0**.**001**
AMS	20 (27.8)	14 (22.9)	6 (54.5)	0.061
Vomiting	18 (25.0)	16 (26.2)	2 (18.2)	0.570
Diarrhea	13 (18.1)	12 (19.7)	1 (9.1)	0.401
Poor feeding	7 (9.7)	7 (11.5)	0 (0)	0.237
GCS, median (IQR)	14 (11–15)	15 (12–15)	11 (9–15)	**0**.**041**
Clinical findings, *n* (%)				
Appearance				
Normal	8 (11.1)	8 (13.1)	0 (0)	0.396
Toxic	64 (88.9)	53 (86.9)	11 (100)	
Skin				
Normal	8 (11.1)	7 (11.5)	0 (0)	0.444
Cutis marmaratus	63 (87.5)	53 (86.9)	10 (80.9)	
Flash	1 (1.4)	1 (1.6)	1 (9.1)	
Pulses				
Normal	18 (25)	16 (26.3)	2 (18.2)	0.763
Weak	53 (73.6)	44 (72.1)	9 (27.9)	
Leaping‐strong	1 (1.4)	1 (1.6)	0 (0)	
Tachycardia	65 (90.3)	58 (95.1)	7 (63.6)	
Bradycardia	3 (4.2)	1 (1.6)	2 (18.2)	
Underlying chronic diseases, *n* (%)				
None	**27 (37**.**5)**	**16 (26**.**2)**	**11 (100)**	**0**.**005**
Malignancy	13 (18.1)	11 (18.0)	2 (18.2)	0.617
CLD	8 (11.1)	7 (11.5)	1 (9.1)	0.921
BMT	5 (6.9)	4 (6.6)	1 (9.1)	0.761
Immune deficiency	11 (15.3)	7 (11.5)	4 (36.4)	**0**.**034**
Cerebral palsy	6 (8.3)	5 (8.2)	1 (9.1)	0.921
SOT	10 (13.9)	9 (14.8)	1 (9.1)	0.325
NMD	6 (8.3)	5 (8.2)	1 (9.1)	0.921
Other	9 (12.5)	7 (11.5)	2 (18.2)	0.368

*Note*: The bold values signify main sentence for characteristics and mean valuable for *p* values.

Abbreviations: AMS, altered mental status; BMT, bone marrow transplantation; CF, cystic fibrosis; CLD, chronic lung disease; CRT, capillary refill time; F, female; GCS, Glasgow Coma Scale; IQR, interquartile range; M, male; min, minimum; max, maximum; NMD, neuro‐metabolic disease; SOT, solid organ transplantation.

^a^ Mann–Whitney *U* and chi‐square tests were used for comparison.

The control group data were as follows: median age 3.0 years (range, 1 month–max 17.0 years, IQR 11 months—8.0 years) and the male:female ratio was 1.2:1. The age and gender of the patient and control groups were similar, and there was no statistically significant difference between the two groups. More than half of the patients (55.6%) were admitted to the ED outside of working hours, and 59.7% had underlying chronic diseases.

### 3.2 Clinical Characteristics and Diagnoses

The most common main complaints were fever (81.9%), difficulty breathing (30.6%), and altered mental status (AMS) (27.8%) (Table 1). Tachycardia (90.3%), toxic appearance (88.9%), cutis marmorata (87.5%), and weak pulses (73.6%) were the most common clinical findings upon admission to the ED. The median Glasgow Coma Scale (GCS) score was 14 (range, 3–15; IQR, 11–15) (Table 1).

The sources of infection were pneumonia (33.3%), bacteremia (27.8%), gastrointestinal system infection (16.7%), catheter infection (9.7%), meningitis (5.6%), pyelonephritis (4.2%), and meningococcemia (2.8%).

On the basis of the initial assessment in the ED, 15 (20.8%) patients were diagnosed with sepsis, 21 (29.2%) with severe sepsis, and 36 (50.0%) with septic shock (Figure 1, Table 1). Overall, AMS was present in 51.4% of patients and was more frequently observed in the severe sepsis (13/21) and septic shock (21/36) groups (*p* = 0.04).

### 3.3 Levels of suPAR for Distinguishing Sepsis, Severe Sepsis, and Septic Shock

**FIGURE 2:**
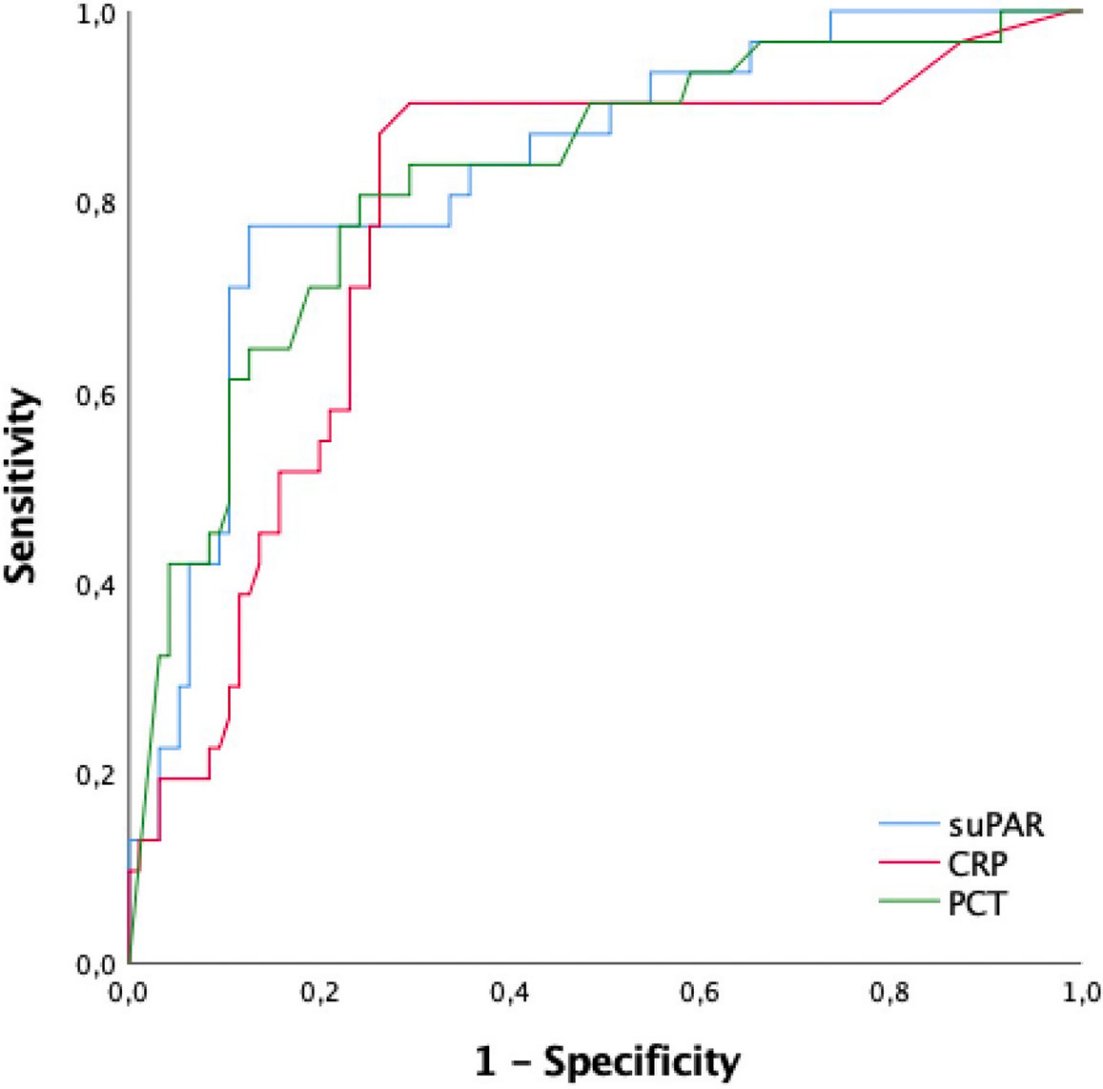
The AUC under the ROC curve of CRP, PCT, and suPAR biomarkers in distinguishing septic shock from sepsis and severe sepsis. The area underneath the ROC curves (AUROCs) were 0.715 (95% CI: 0.583–0.846), 0.520 (95% CI: 0.376–0.663), and 0.642 (95% CI: 0.504–0.779) of suPAR, CRP, and PCT (*p =* 0.521, 0.852, and 0.623, respectively).

Hematological test findings, CRP, PCT, and plasma suPAR levels of patients and control groups are shown in Table 2. Plasma suPAR levels were significantly higher in sepsis, severe sepsis, and septic shock groups than in the control group (*p* < 0.001, *p* < 0.001, and *p* < 0.001) (Table 2). Patients in the septic shock group had the highest suPAR levels on admission to the ED when compared with the sepsis and severe sepsis groups (*p* = 0.009 and 0.042, respectively) (Table 2). The area underneath the ROC curve (AUROC) of plasma suPAR, CRP, and PCT biomarkers for septic shock is shown in Figure 2. It was observed that AUROC for PCT, CRP, and suPAR values did not exhibit statistically significant differences. When the suPAR results were analyzed using the ROC curve method, the optimum diagnostic cutoff point for septic shock was 4.5 ng/mL, the AUROC was 0.715 (95% CI: 0.583–0.846), sensitivity 77.4% (95% CI: 74–88%), specificity 87.4% (95% CI: 81–96%), PPV 68.3%, and NPV 91.4% (Figure 2, Table 3). Also, when comparing the data of patients and the control group, a plasma suPAR level of ≥4.5 ng/mL can be utilized to predict septic shock (OR 9.1, 95% CI 3.1–26.6).

**TABLE 2:** Comparison of hematological tests, CRP, PCT, and suPAR levels between the patient groups and mortality.

	WBC (/mm^3^)	ANC (/mm^3^)	CRP (mg/dL)	PCT (μg/L)	suPAR (ng/mL)
Patients, median (IQR)	11,120 (16435–19,170)	10,745 (3700–14,680)	7.5 (1.1–14.0)	6.2 (0.6–79.7)	4.8 (2.6–12.1)^a^
Sepsis	14,670 (8890–20,890)	8240 (5930–16,200)	8.5 (3.6–15.2)	1.3 (0.4–5.8)	3.5 (1.4–8.6)^a^
Severe sepsis	8430 (5490–16,840)	6090 (2870–12,460)	6.1 (0.3–14.1)	6.1 (1.7–30.8)	3.9 (1.2–15.2)^a^
Septic shock	10,690 (6280–20,710)	8290 (4160–15,460)	6.6 (1.2–13.9)	22.0 (1.9–100.0)	7.6 (1.6–44.3)^a^, ^b^
Controls median (IQR)	8235 (6625–10,203)	2570 (2160–3510)	0.3 (0.2–0.4)	0.1 (0.1–0.2)	2.0 (1.5–3.0)
Mortality, median (IQR)					
Survivors	10,040 (6120–18,570)	8010 (2690–14,320)	7.8 (1.2–13.9)	6.6 (0.6–91.9)	4.2 (1.2–24.8)
Nonsurvivors	12,250 (7960–25,110)	9130 (4540–15,580)	8.6 (0.3–19.1)	2.3 (0.2–8.0)	9.7 (7.5–12.3)^c^

*Note:* ANOVA test was used for the comparison of sepsis, severe sepsis, and septic shock groups.

Abbreviations: ANC, absolute neutrophil count; CRP, C‐reactive protein; IQR, interquartile range; PCT, procalcitonin; suPAR, soluble urokinase plasminogen activator receptor; WBC, white blood cell.

^a^ Plasma suPAR levels were significantly higher in sepsis, severe sepsis, and septic shock groups than the control group (*p* < 0.001, *p* < 0.001, and *p* < 0.001, respectively).

^b^ Plasma suPAR levels were significantly higher in the septic shock group on admission to the ED than in the sepsis and severe sepsis groups (*p* = 0.009 and 0.042, respectively).

^c^ Nonsurvivors demonstrated markedly higher suPAR levels in comparison with survivors (*p* < 0.001).

**TABLE 3:** Sensitivity (%), specificity (%), PPV (%), and NPV (%) of CRP, PCT, and suPAR levels on the admission in septic shock and mortality.^a^

	Septic shock					Mortality				
	Sensitivity (%)	Specificity (%)	YI	PPV (%)	NPV (%)	Sensitivity (%)	Specificity (%)	YI	PPV (%)	NPV (%)
CRP (mg/dL)										
≥8	50.0	61.5	0.12	50.0	82.8	50.0	56.5	0.10	45.5	50.8
PCT (μg/L)										
≥2	73.3	42.3	0.16	68.8	77.3	70.0	34.8	0.15	63.6	32.7
SuPAR (ng/mL)										
≥4.5	77.4	87.4	0.65	68.3	91.4	100	58.5	0.32	28.9	100
≥7.0	61.1	80.6	0.42	68.8	86.3	91.0	87.4	0.78	90.9	97.9

Abbreviations: CRP, C‐reactive protein; PCT, procalcitonin; suPAR, soluble urokinase plasminogen activator receptor; PPV, positive predictive value; NPV, negative predictive value; YI, Youden index.

^a^ Receiver operating characteristic (ROC) analysis was utilized for significant variables to calculate cutoff points, with sensitivity and specificity, PPV, and NPV.

### 3.4 Comparison of Plasma suPAR for Predicting Mortality From Sepsis and/or Septic Shock

**FIGURE 3:**
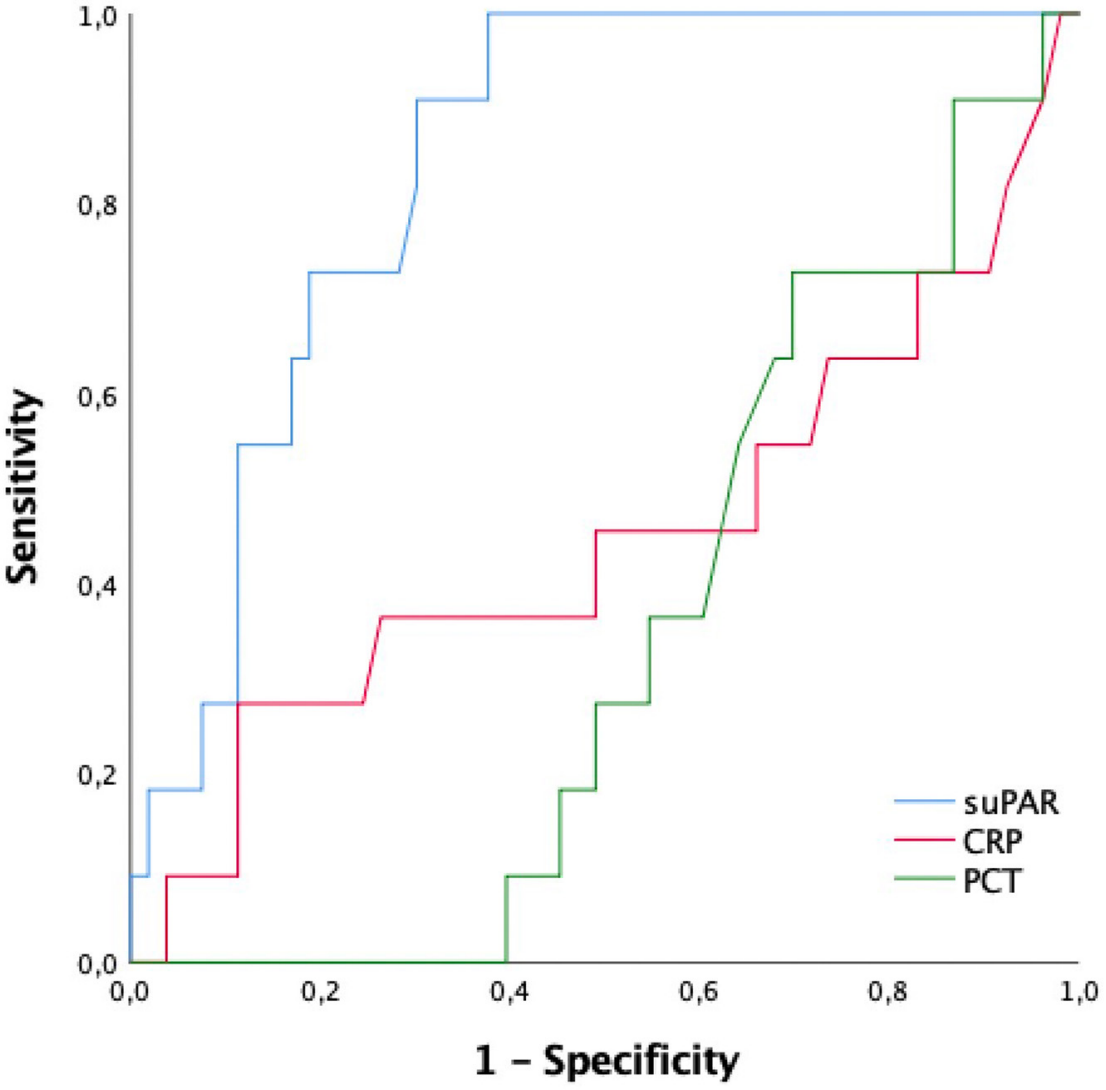
The AUC under the ROC curve of plasma suPAR, CRP, and PCT biomarkers for mortality. The area underneath the ROC curves (AUROCs) were 0.840 (95% CI: 0.739–0.940), 0.449 (95% CI: 0.234–0.665), and 0.346 (95% CI: 0.204–0.489) of suPAR, CRP, and PCT (*p* = 0.962, 0.021, and 0.002, respectively).

The AUROC of plasma suPAR, CRP, and PCT biomarkers for mortality is shown in Figure 3. The AUROC for plasma suPAR was significantly higher than the AUROC of CRP and PCT (*p* = 0.021 and 0.002). When the suPAR results were analyzed using the ROC curve method, the optimum diagnostic cutoff point was 7.0 ng/mL, the AUROC was 0.840 (95% CI: 0.740–0.937), sensitivity 91.0% (95% CI: 82–98%), specificity 87.4% (95% CI: 78–96%), PPV 90.9% and NPV 97.9% (Table 3). Plasma suPAR demonstrated the highest sensitivity, PPV, and NPV in predicting mortality.

Univariate and multivariate logistic regression analyses were performed to identify predictive biomarkers and other factors for mortality (Table 4). There was no observed disparity in CRP and PCT levels between survivors and nonsurvivors upon admission to the ED (Table 4). Nonsurvivors demonstrated markedly higher suPAR levels in comparison with survivors (9.7 ng/mL vs. 4.2 ng/mL) (*p* < 0.001) (Table 2).

**TABLE 4:** Predictor factors of mortality on admission to the ED.^a^

	Survivor/nonsurvivor (%)	Univariate			Multivariate		
		OR	95% CI	*p*	OR	95% CI	*p*
Gender							
Male (R)	30/6						
Female	31/5	0.8	0.2–2.9	0.744	—	—	—
Age (years)							
>1 (R)	46/6						
≤1	15/5	0.4	0.1–1.5	0.164	—	—	—
Admission time							
W (R)	27/5						
OW	34/6	0.9	0.3–3.5	0.942	—	—	—
Underlying disease							
No (R)	27/0						
Yes	34/11	4.7	1.1–9.0	**<0**.**001**	6.8	2.3–8.9	**<0**.**001**
CRP (mg/dL)							
<8 (R)	30/6						
≥8	31/5	1.0	0.2–4.4	0.987	—	—	—
PCT (μg/L)							
<2 (R)	18/4						
≥2	35/7	0.6	0.1–3.0	0.562	—	—	—
SuPAR (ng/mL)							
<7 (R)	49/4						
≥7.0	12/7	9.3	2.1–40.2	**0**.**003**	17.2	2.4–61.3	**0**.**006**
pSOFA							
<5	22/1						
≥5	39/10	0.2	0.1–1.4	0.110	3.9	0.4–34.9	0.217
PICU admission							
+ (R)	24/8						
−	37/3	4.1	0.9–17.1	0.051	0.4	0.08–2.1	0.275

*Note*: The bold values signify main sentence for characteristics and mean valuable for *p* values.

Abbreviations: CRP, C‐reactive protein; PCT, procalcitonin; PICU, pediatric intensive care unit; pSOFA, pediatric sequential organ failure assessment; R, reference; suPAR, soluble urokinase plasminogen activator receptor; OR, odds ratio; CI, confidence interval.

^a^ Univariate and multivariate logistic regression analyses.

A plasma suPAR level ≥7.0 ng/mL can be utilized to predict mortality in patients with septic shock (OR 17.2, 95% CI 2.4–61.3) (*p* = 0.006) (Table 4).

### 3.5 Outcomes

More than half of the patients (55.5%) were admitted to the ward, and the remaining patients were admitted to the PICU (Figure 1). Patients with severe sepsis and septic shock receive their initial interventions and management in the ED. During this phase, some patients with severe sepsis may exhibit clinical improvement, and those managed for septic shock in the ED may experience resolution of shock and subsequently be admitted to the general ward. However, patients who fail to respond to treatment, deteriorate into severe sepsis or progress to septic shock, or do not respond to treatment while in septic shock, are admitted to the PICU. Also, the plasma suPAR levels upon admission of patients to the PICU were statistically significantly higher than those admitted to the ward (median 7.4 ng/mL vs. 3.5 ng/mL, respectively) (*p* = 0.004). The overall mortality rate was 15.3% (11/72), and three patients died within 24 h of admission to the ED. Two died on Day 1–Day 2 (D1–D2), 2 on D2–D4, 2 on D4–D7, and 2 on D7–D28 in PICU (Table 5). Although the mortality rate among patients admitted to the PICU was observed to be higher than those admitted to the ward (33.0% vs. 8.1%, respectively), there was no statistically significant difference (*p* = 0.052). In patients with observed mortality, there was a presence of chronic illness. The presence of a chronic condition in children with sepsis significantly increased the risk of mortality (OR 6.8, 95% CI 2.3–8.9, *p* < 0.001) (Table 4).

**TABLE 5:** Demographic characteristics and suPAR values of nonsurvivors at admission to the ED.

No.	Gender	Age (year)	Admission time	suPAR (ng/mL)	Mortality time (h)	Underlying chronic diseases
1	F	12	OW	9.76	23	Immune deficiency
2	F	6.5	W	5.01	92	Rett syndrome
3	M	1	W	8.80	40	Joubert syndrome
4	M	8	W	9.75	672	Leukemia, BMT
5	F	1	OW	12.28	8	Dilated CMP
6	M	12	W	9.75	5	Cerebral palsy
7	M	14	OW	7.48	80	Friedreich ataxia, hypertrophic CMP
8	M	6 months	OW	24.50	696	Down syndrome, AVSD
9	F	8	OW	9.26	34	Chediak–Higashi syndrome
10	M	6 months	W	44.32	144	Ehler–Danlos syndrome
11	F	7 months	OW	7.25	156	Krabbe disease

Abbreviations: AVSD, atrioventricular septal defect; BMT, bone marrow transplantation; CMP, cardiomyopathy; ED, emergency department; F, female; M, male; OW, out of working; suPAR, soluble urokinase plasminogen activator receptor; W, working.

## 4 Discussion

This study provides valuable insights into the clinical characteristics, diagnosis, and outcomes of pediatric patients with sepsis and/or septic shock. Additionally, it highlights the potential of plasma suPAR levels as diagnostic and prognostic markers for identifying septic shock and predicting mortality in the pediatric population.

The role of conventional inflammatory biomarkers (CRP and PCT) in the diagnosis of sepsis/septic shock and the prediction of prognosis is controversial [16, 17, 18, 19, 20]. Also, these biomarkers are mostly increased in severe bacterial infections, but their late rise, elevation during some viral infections, and the requirement for serial measurement limit their use in the ED for the differential diagnosis and prognosis of sepsis and septic shock [20, 21, 22].

The results of several studies investigating suPAR levels in sepsis are inconsistent [23, 24]. Although plasma suPAR levels increase in the conditions mentioned above, Wittenhagen et al. determined suPAR levels in healthy children to be 2.3 ng/mL [25]. The levels of suPAR in control healthy children were found to be consistent with the literature (2.0 ng/mL). Although there was no significant difference in suPAR levels between the sepsis and severe sepsis groups, the suPAR levels in children with sepsis were higher than healthy children (3.5 ng/mL vs. 2.0 ng/mL). Inconsistent with previous studies, our results also indicate that suPAR concentrations reflect the severity of the infection and are associated with a worse outcome. The highest suPAR levels were found in children with septic shock. Assuming suPAR to be a prognostic marker in septic shock would facilitate the implementation of evidence‐based lifesaving therapeutic interventions, such as rapid fluid resuscitation, antimicrobial treatment, central venous pressure monitoring, and vasopressor usage, leading to a direct decrease in mortality rates.

Septic adult patients exhibit threshold values of suPAR, but there are insufficient data on children with sepsis. According to Khater et al., in elderly patients who admitted to ICU, the sensitivity and specificity for sepsis were 97.5% and 90%, respectively, if the suPAR level was ≥4.37 ng/mL [26]. Prior research has demonstrated a significant connection between raised suPAR levels and mortality caused by sepsis in adults [27, 28]. Nevertheless, a methodological disparity was evident in our study compared with previous research, wherein the assessment of suPAR levels in critically ill patients was conducted postadmission, several days after ICU admission, rather than at the time of initial presentation. In their study, Ostrowski et al. analyzed suPAR levels in children with malaria. They found that suPAR levels were higher in nonsurvivors or those with complicated malaria. Additionally, the mortality rate increased in children whose suPAR values exceeded 1 ng/mL [29]. This study found that suPAR levels of 4.4 ng/mL or higher predict septic shock in children. Furthermore, a suPAR cutoff value of 7.0 ng/mL or higher had a sensitivity, specificity, PPV, and NPV for mortality of 91%, 77%, 90.9%, and 97.9%, correspondingly. These outcomes indicate a correlation between elevated suPAR levels and mortality in children with sepsis. When suPAR values were less than 7 ng/mL, the survival rate was significantly better.

The mortality rate of sepsis can differ on the basis of the quality of care in pediatric intensive care units (PICUs) and the socioeconomic status of patients in the country [30]. According to Schlapbach et al., the rate of sepsis‐related mortality was 8.5% in New Zealand and Australia, 1.3% in Spain, 3.5% in China, 5.4% in Canada, 8.9% in the USA, and 16.6% in Germany [5, 31, 32, 33, 34, 35]. In this study, the rate of mortality was 15.3%. All nonsurvivors in our study suffered from at least one chronic underlying disease. We have a 16‐bed pediatric intensive care unit that offers the most advanced medical care to critically ill children. Despite the advanced and excellent care provided in our center's PICU, the rate of mortality was higher than what has been reported in the literature. The possible cause of this variation may be the concurrent prevalence of chronic illnesses, which in related research has been notably linked to an increased risk of mortality associated with sepsis [36].

The study offers valuable insights into pediatric patients with sepsis and septic shock and examines the potential of plasma suPAR levels as a diagnostic and prognostic marker. However, it also has some limitations that need to be considered: (1) The study was conducted at a single center, which could restrict the general applicability of the findings to a broader population; conducting multicenter studies in diverse settings could substantiate the results; (2) the research does not thoroughly address or correct for possible confounding variables that may impact the link between suPAR levels and clinical outcomes; (3) when this study was conducted, the patient groups were formed according to the classification “sepsis, severe sepsis, and septic shock” in the International Consensus Conference on Pediatric Sepsis definition; and (4) the majority of studies in the literature comparing our study's suPAR level results primarily focused on adult patients admitted to the ICU with diagnosed sepsis or septic shock. Consequently, our study presents methodological differences because of these variations in patient populations compared with the referenced literature. Even with these limitations, the research contributes valuable information to the current understanding of pediatric sepsis and septic shock while emphasizing the possible role of plasma suPAR levels as a diagnostic and prognostic marker. Further research with bigger sample sizes, prospective designs, and external validation is necessary to improve the evidence and offer more reliable insights into the usefulness of suPAR in this clinical context.

## 5 Conclusion

Plasma suPAR levels have the potential as a biomarker for predicting mortality in pediatric septic shock patients. Also, in children with septic shock, the presence of plasma suPAR ≥7 ng/mL along with an underlying disease significantly increases the risk of mortality. These discoveries may have implications for enhancing early treatment and predicting the prognosis of septic shock and pediatric sepsis, which may ultimately lead to better outcomes.

## Conflicts of Interest

The authors declare no conflicts of interest.

## Supporting information


Table S1.

## Data Availability

The data that support the findings of this study are available from the corresponding author upon reasonable request.
