# Supplementary material for: The Role of Soluble Urokinase Plasminogen Activator Receptor (suPAR) as an Early Indicator of Mortality in Pediatric Septic Shock
Source: J Clin Lab Anal. 2024 May 6;38(9):e25040. doi: 10.1002/jcla.25040 (PMC11137844; doi:10.1002/jcla.25040)
Supplement: Supplementary file 1 — Table S1. [file JCLA-38-e25040-s001.docx]

Supplementary table. Pediatric Sequential Organ Failure Assessment Score (pSOFA)

|  | **0** | **1** | **2** | **3** | **4** |
| --- | --- | --- | --- | --- | --- |
| **Respiratory**   - PaO_2_ / FiO_2_   or   - SaO_2_ / FiO_2_ | ≥ 400  ≥ 292 | 300-399  264-291 | 200-299  221-264 | 100-199 (+MV)  148-220 (+MV) | < 100 (+MV)  < 148 (+MV) |
| **Coagulation**   - Platelet count (x10^3^/μL) | ≥ 150 | 100-149 | 50-99 | 20-49 | <20 |
| **Hepatic**   - Bilirubin (mg/dL) | < 1.2 | 1.2-1.9 | 2.0-5.9 | 6.0-11.9 | >12.0 |
| **Cardiovascular**  [MAP (mm Hg) or  inotrop use (μg/kg/m)]   - < 1 mo - 1-11 mo - 12-23 mo - 24-59 mo - 60-143 mo - 144-216 mo - >216 mo | ≥ 46  ≥ 55  ≥ 60  ≥ 62  ≥ 65  ≥67  ≥70 | < 46  < 55  < 60  < 62  < 65  < 67  <70 | Dopamin  (≤5) ya da  Dobutamin | Dopamin (>5) /  Epinefrine ≤0.1 /  Norepinefrine ≤0.1 | Dopamin (>15)  Epinefrine > 0.1  Norepinefrine > 0.1 |
| **Neurologic**   - GCS | 15 | 13-14 | 10-12 | 6-9 | <6 |
| **Renal** (Creatinin, mg/dL)   - < 1 mo - 1-11 mo - 12-23 mo - 24-59 mo - 60-143 mo - 144-216 mo - >216 mo | < 0.8  < 0.3  < 0.4  < 0.6  < 0.7  < 1.0  < 1.2 | 0.8-0.9  0.3-0.4  0.4-0.5  0.6-0.8  0.7-1.0  1.0-1.6  1.2-1.9 | 1.0-1.1  0.5-0.7  0.6-1.0  0.9-1.5  1.1-1.7  1.7-2.8  2.0-3.4 | 1.2-1.5  0.8-1.1  1.1-1.4  1.6-2.2  1.8-2.5  2.9-4.1  3.5-4.9 | ≥1.6  ≥1.2  ≥1.5  ≥2.3  ≥2.6  ≥4.2  ≥5 |

FiO_2_: fraction of inspired oxygen; MAP: mean arterial pressure; SpO_2_: peripheral oxygen saturation; MV: mechanic ventilation; GCS: Glasgow coma scale; mo: month
